# The Relationship Between Commuting Stress and Nurses' Well-Being: Considering Gender Differences

**DOI:** 10.1155/jonm/4414417

**Published:** 2025-02-25

**Authors:** DanYang Li, Jeffery D. Houghton, Xuan Li, QiQi Peng, JianQing Li, WenChi Zou

**Affiliations:** ^1^School of Business, Macau University of Science and Technology, Macau, China; ^2^John Chambers College of Business and Economics, West Virginia University, Morgantown, West Virginia, USA; ^3^The Third Affiliated Hospital of Guangzhou Medical University, Guangzhou, China; ^4^Shenzhen Nanshan Maternity and Child Healthcare Hospital, Shenzhen, China

**Keywords:** commuting stress, emotional exhaustion, gender, nurses' well-being

## Abstract

**Aim:** This research explores how and why commuting stress influences Chinese nurses' well-being.

**Background:** A daily work commute may result in a significant psychological risk factor that can lead to harmful physiological and mental health consequences. Emerging research indicates that nurses experience long-lasting negative effects on their mental health, including burnout, due to the stress of a daily work commute.

**Methods:** The study employed a cross-sectional design involving 380 registered nurses from state-owned hospitals in southern China. Hypotheses were tested using the PROCESS macro developed by Hayes [1].

**Results:** The stress of commuting indirectly influences nurses' well-being through increased emotional exhaustion. There is an interaction between commuting stress and nurses' gender such that the indirect effect of commuting stress on well-being is stronger for female nurses.

**Conclusions:** The spillover effect of commuting stress in the nursing profession is often underestimated. However, this study underscores its significant impact on nurses' emotional exhaustion and well-being. The research findings emphasize that commuting stress contributes to emotional exhaustion and a decline in nurses' well-being.

**Implications for Nursing Management**: Healthcare organizations should leverage these findings to reinforce the importance of self-care for nurses and to provide nurses with resources to help decrease the potential negative outcomes of commuting stress. The study also shows that female nurses may experience a more pronounced association between commuting stress and emotional exhaustion. As such, healthcare organizations should develop gender-specific interventions and support systems that address the unique challenges faced by female nurses in relation to commuting stress.

## 1. Introduction

Commuting is a critical modern phenomenon that is a function of the spatial interaction between employee housing and labor markets. Against this social background, many people commute a long distance from their home to their workplace, especially in megalopolises [2–4]. Scholars have argued that a daily work commute may be an important psychological risk factor which could elicit unhealthy physical and mental states [2, 5]. Moreover, scholars also suggest that nurses, in particular, may experience enduring negative impacts from commuting stress, such as burnout, on their mental health [6–8]. Consequently, daily commuting represents a new and underexplored potential stressor and burden for nurses [6, 7].

Within the past twenty years, interest in understanding how workplace conditions as they relate to nurses' mental health issues has experienced increased attention. The focus of this interest is primarily regarding the issues of mental health, burnout, and emotional well-being [9, 10]. Using the job demands–resources (JD-R) model, researchers have sought to clarify that the issue of burnout can be described on a scale rather than being a fixed state of being [9, 11]. Application of the JD-R model to the nursing environment reveals how the nursing practice itself, long hours, working with patients whose needs include emotional support, etc., produces a unique cocktail of exhaustion leading to declining engagement with their work [11]. It comes as no surprise then that measurements of emotional exhaustion can be predictive as a first step toward burnout [9, 12]. While the nature of nursing creates demands on the nurses' emotional reserves, the work is not the only factor leading to exhaustion. Stressors outside the workplace may also contribute to the decline of a nurse's mental health and well-being [2, 4, 12]. For instance, scholars have argued that commuting spillover is a significant antecedent that may have a direct impact on an individual's psychological states (e.g., flow experience and well-being), behaviors (e.g., job performance), and cognition (e.g., intention to quit) [2]. The current study employs both the JD-R model and conservation of resources theory (COR) to explore how commuting spillover may serve as a nonwork contextual factor that may precipitate nurses' emotional exhaustion.

As noted above, interest in the well-being of nurses continues to grow [13–16]. A primary reason for this burgeoning interest is that healthcare organizations strive to enhance the health levels of society as a whole, and the well-being of healthcare workers (e.g., nurses) is strongly linked to patient well-being because workers with high levels of well-being are more likely to deliver high quality care, which can influence patient well-being [17]. Meanwhile, the nursing profession is facing significant workforce shortages globally [18] in combination with high nurse turnover rates [19], resulting in adverse medical conditions in many nations [20, 21]. Emotional exhaustion is a significant contributing factor to nurses leaving the profession or experiencing a sense of low job well-being [9, 21]. Prior research results reveal that emotionally exhausted nurses are more likely to experience reduced empathy, decreased job performance and career satisfaction, increased medical errors, and intention to leave the profession, etc. [10]. These findings imply that nurses' emotional exhaustion has not only a negative direct impact on the nurses themselves but also adverse effects on patient care and well-being. Therefore, studying the impact of emotional exhaustion on nurses' well-being can provide evidence-based recommendations for retaining nurses and decreasing turnover rates.

**Figure 1:**
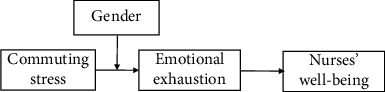
Hypothesized model.

The purpose of the present study is to probe the relationship between nurses' commuting stress and well-being as mediated by emotional exhaustion as conditional on individual differences (i.e., gender). Prior studies have revealed that even as women's involvement in the labor market has grown in China, a gender gap in commuting has largely endured, similar to the situation in Western developed countries [22, 23]. According to the gender role perspective, Chinese female nurses are expected to undertake greater household and childcare responsibilities, which impose constraints on Chinese female nurses' time and resources (e.g., physical energy and patience) that make them eager to spend less time and resources in commuting to work [22, 24]. Moreover, studies have indicated that women are more inclined to use public transportation, which brings more stressful experiences, rather than private automobiles for their daily commute, compared to men [22, 25]. Therefore, this study employs gender role theory to probe whether the gender of Chinese nurses as individual differences will strengthen or weaken the relationship between nurses' commuting stress and their emotional exhaustion. In short, our study advances the nursing commuting literature by incorporating social role theory and COR theory to examine nurses' gender as a moderating mechanism to help explain the association between nurses' commuting stress and emotional exhaustion.  Figure 1 exhibits our conceptual framework, which we explain in detail below.

## 2. Theory and Hypothesis

### 2.1. Commuting Stress and Emotional Exhaustion

The COR theory proposes that individuals are motivated to acquire, maintain, enhance, and safeguard resources that they consider highly valuable [26]. Within this perspective, nurses are likely to rely on certain essential resources for self-regulation, managing their social interactions, and aligning their behavior with hospital and professional expectations [26]. According to the COR theory, nurses experience increased stress and decreased well-being when their resources become depleted [27–29]. Hobfoll [28] defines the concept of “resources” within the COR framework comprehensively, including various items, personal traits, conditions, or forms of energy that possess either inherent or transactional value. If nurses can restore or regenerate the resources expended in confronting stress, adverse consequences can be averted as long as the rate of restoration keeps pace with the rate of depletion. However, when resource depletion outpaces renewal, nurses face increasing stress and strain [29], which can compromise their well-being over time [30, 31].

The commute to work is frequently regarded as a crucial component in enabling nurses to fulfill their professional roles, as it facilitates the transition from their private life to their occupational responsibilities, allowing them to mentally prepare for workplace tasks, duties, and interactions. According to the JD-R model, any job element that facilitates the attainment of occupational goals is considered a resource, while elements that hinder these goals constitute demands [11]. A challenging commuting experience constitutes a demand that may deplete nurses' personal resources, notably in terms of time and energy, prior to their arrival at the workplace. For those nurses who face a difficult or depleting commute, a direct link can be drawn highlighting the impact on their emotional well-being and proclivity toward burnout [2, 4].

According to the principles of the COR theory, resources available to a nurse include time, physical energy, and psychological assets for emotional self-management. When one or more of these resources are depleted or insufficiently restored in a suitable time frame, stress in the nurse will increase [29]. An example of this dynamic is seen in the stress induced by a difficult commute to work. A long or difficult commute consumes the nurse's time and requires physical energy and emotional resources to be spent without concurrent replenishment such as a sense of satisfaction they may find through work or leisure activities [2, 4]. Consider the impact of a daily commute: crowded trains or buses present nurses with conditions of stress upon their personal space. Driving a private car introduces the stress of navigating traffic congestion, potential road rage, and time issues. These conditions can produce physical and psychological fatigue in the nurse [2, 3]. The stress of a daily commute may be most directly felt as the nurse faces a depletion of the time resource often in ways beyond their control to change. For instance, delays due to poor weather, accidents, road construction, or other unforeseen reasons rob the nurse's time. These conditions may also result in the nurse arriving late to work, creating even more stress [2, 3]. A lengthy or difficult commute only adds stress to what is an otherwise long work schedule for nurses creating heightened potential for their slides toward exhaustion and burnout [9]. Therefore, we argue the following hypothesis.

**Hypothesis 1 .**

Commuting stress is positively associated with nurses' emotional exhaustion at work.

### 2.2. Emotional Exhaustion and Nurses' Well-Being

Scholars have argued that the definition of individual well-being in the workplace is characterized by a shared understanding but remains imprecise [32, 33]. More precisely, prior studies on individual well-being in the workplace have evolved from a broader understanding of general well-being, encompassing both subjective well-being and psychological well-being (PWB) [33–35]. To date, because individual studies have developed separate theoretical frameworks for exploring employee well-being, there is a persistent lack of a standardized definition and consistent measurement methods for studying individual well-being in the workplace [33]. Furthermore, cultural disparities between Eastern and Western societies result in distinct perspectives on well-being held by the Chinese population. For example, the Chinese collectivistic culture places emphasis on harmony, societal orientation, and an interdependent self [33, 36]. Given this context, the present study adopts Zheng et al.'s [33] comprehensive well-being framework, which proposes that nurses' well-being encompasses workplace well-being (WWB), PWB, and life well-being (LWB). This multidimensional approach facilitates a more holistic assessment of Chinese nurses' well-being.

Emotional exhaustion is particularly relevant in the context of the COR theory, as it represents a form of resource depletion [29, 37, 38]. It is characterized as one of the typical symptoms of nurse burnout, the other two being depersonalization and decreased personal accomplishment [10]. This phenomenon arises from the constant and chronic occupational stress [39] that is a prominent characteristic of nursing [40]. Research has shown that emotional exhaustion is a primary driver of a burnout episode, exhibiting the most consistent relationship within its nomological network [37, 38].

Nurses experiencing emotional exhaustion are likely depleting psychological resources at a faster rate than they can replenish them. This is due to the high job demands of the nursing profession, including long shifts, dealing with life-and-death situations, high patient loads, the emotional demands of caring for sick and dying patients, experiencing posttraumatic stress disorder, and navigating bureaucratic pressures [9, 10, 40]. Consequently, nurses become trapped in a cycle of resource loss that can be challenging to break. This cycle of resource loss, by way of either potential or actual losses, then results in intensifying the positive feedback nature of exhaustion both psychically and physically [29]. Once emotional exhaustion sets in, the nurses' resources for coping with stress face additional depletion as their ability to handle the demanding nature of their work diminishes [41]. One result of this downward spiral is a loss in enthusiasm for the work itself, low morale, potentially increased absenteeism, and compassion fatigue. In other words, burnout results [12, 41].

The downward, positive feedback loop of resource depletion saps the nurse's ability to deal with stress and erodes resiliency. At the lowest point, the nurse may suffer from anxiety, depression, or other psychologically negative conditions [40, 42]. With their decreased resiliency and overall fatigue setting in, a nurse is then more prone to be inattentive, forgetful, and decision fatigue. The resulting errors in judgment and practice will only further the decline in a nurse's sense of competence and well-being [9, 13], bringing them to the point of burnout if interventions are not taken to help.

It is not unexpected to find a nurse experiencing well-being issues outside of work times as the stress from work spills over into their private lives [2]. Decreased well-being in the workplace results in a decrease in energy and passion for even the enjoyable and replenishing aspects of time outside work. To make matters worse, some nurses overconsume caffeinated beverages, take up smoking, or find other unhealthy strategies for avoiding their exhaustion [43–45]. These unhealthy strategies of avoidance only increase the downward trajectory of their sense of well-being and life satisfaction. On the basis of these logical and theoretical arguments, we contend the following hypothesis.

**Hypothesis 2 .**

Emotional exhaustion is negatively associated with nurses' well-being.

### 2.3. Commuting Stress and Nurses' Well-Being

According to the COR theory, all workers seek to protect their pool of resources and nurses are no different [29]. The stress associated with commuting to work is a resource drain and can add to the downward spiral of a nurse's sense of well-being [2, 4, 46]. One way a long commute depletes a nurse's resources is by limiting their opportunity for physical exercise or additional professional training. The time spent commuting also adversely affects a nurse's social life in and out of work, thus decreasing their sense of communal belonging and well-being [2]. Other factors affected by the commute include a depletion of the nurse's thought processes and the ability to tolerate and cope with life issues [47]. The negative aspects of commuting to work force the nurse to use additional personal energy and time resources that are then not available to their patients at work, again reducing their capacity for empathy and readiness to engage in the nursing tasks [2, 47]. Commuting is not only a psychological drain as the process of the commute requires additional cognitive resources to be spent, again depleting the nurse's resources for a job requiring creativity, problem-solving, and a proactive mindset [2, 48]. When a nurse faces a regular, stressful commute scenario, he or she may develop a persistent sense of heightened anxiety and/or irritability. These compounding stressors result in a condition of overload such that the nurse enters a state of overall negativity toward her or his work and life.

According to research based on the JD-R model, high job demands combined with low job control can lead to increased stress and less healthy outcomes for nurses [13, 49–51]. Rather than viewing the commute as an external issue, recognizing the commute to be a work-related demand adds to the sense of low job control [2, 6]. As highlighted earlier, a work commute confronts the nurse with stressors such as traffic congestion, long travel times, demanding schedules, and the like. These issues further add to the nurse's lack of control and bring about increased stress and a decreased sense of well-being. In summary, existing theory and empirical research suggest that commuting stress, as an additional job demand for nurses with limited control, can result in detrimental outcomes including heightened stress, reduced cognitive resources, and an overall negative evaluation of well-being at work. Thus, we predict the following hypothesis.

**Hypothesis 3 .**

Commuting stress is negatively associated with nurses' well-being.

Taken together, Hypotheses 1–3 as described above specify a mediation model through which commuting stress indirectly decreases nurses' well-being by contributing to their emotional exhaustion. Accordingly, we add the following hypothesis.

**Hypothesis 4 .**

Emotional exhaustion mediates the negative relationship between commuting stress and nurses' well-being.

### 2.4. The Moderating Effect of Gender

We further propose that gender moderates the linkage between nurses' commuting stress and emotional exhaustion. In accordance with social role theory [52], gender differences arise because of gender roles that are founded on traditional labor divisions, which place women in the roles of homemakers and caregivers and men in the role of breadwinners. Social gender roles and expectations can influence how women and men navigate their professional and personal lives. Women often face additional expectations and responsibilities related to caregiving, household tasks, and family obligations, which can make it more difficult for them to balance work and family responsibilities [22, 53]. Within this social context, research conducted in China has provided strong empirical evidence that women's family roles are associated with gendered commuting characteristics [22, 23, 54]. Already burdened by a challenging professional role and social expectations for household management, the added stress of a commute can be the tipping point into emotional exhaustion.

In addition to the societal stress placed upon female nurses, i.e., domestic expectations and roles, they are less likely to have control over their commute experience due to being more likely to depend on public transport rather than a private vehicle for their commute [22, 25, 55]. When public transportation systems are scarce or inefficient, female nurses may experience increased stress. Additionally, female nurses working shifts that require travel during early or late hours may experience even greater levels of anxiety and stress due to taking on more responsibilities for caring for their families than men do. In summary, the existing literature suggests that the combination of the gendered nature of commuting and the additional responsibilities faced by female nurses may intensify the negative influence of commuting stress on their emotional exhaustion. Consequently, we proffer the following hypothesis.

**Hypothesis 5 .**

The positive association between commuting stress and emotional exhaustion is moderated by gender, such that there is a stronger positive relationship between commuting stress and emotional exhaustion for female nurses than for male nurses.

Taken together, Hypotheses 1–5 imply a moderated mediation model in which the indirect relationship between commuting stress and nurses' well-being is conditionally influenced by gender. Thus, our final hypothesis proposes first-stage moderated mediation.

**Hypothesis 6 .**

Gender moderates the indirect effect of commuting stress on nurses' well-being through emotional exhaustion, with the effect being stronger for female nurses than for male nurses.

## 3. Method

### 3.1. Participants and Procedure

This study used a convenience sample of data from five hospitals in southern China that are operated and owned by the government. The targeted population was frontline registered nurses. To participate in the study, nurses were required to be providing direct care to patients and to have been in their current positions for at least six months.

The study collected data from nurses at three separate time points. Initially, the nurses provided demographic information and their work IDs and assessed their commuting stress and emotional exhaustion. Strict confidentiality was assured. One month later, the nurses completed a second survey assessing their emotional exhaustion. Two months after the initial survey, the nurses completed a third survey reporting their levels of well-being. The temporal separation in data collection helped to mitigate potential common method bias [56]. The final sample included 380 complete responses, which represented 82% of the invited nurses. Participants had an average age of 32 and an average tenure of 9.1 years, with 77% female and 23% male.

### 3.2. Measures

The working IDs of the participants facilitated matching of the multiple surveys. Participants completed the survey items on a Likert-type scale with endpoints spanning from 1 (*strongly disagree*) to 5 (*strongly agree*).

Commuting stress: Commuting stress was assessed using a 10-item scale developed by Amponsah-Tawiah, Annor, and Arthur [57]. An example scale item is “My morning commute to work was taking longer than I expected.”

Emotional exhaustion: Emotional exhaustion was measured using the 5-item scale from Li and Shi [58], which was developed for the Chinese context from the Maslach Burnout Inventory (MBI-D) [59]. An example scale item is “I feel emotionally drained from my work.”

Nurse's well-being: Nurse's well-being was measured by Zheng et al.'s [33] scale, which includes three subscales assessing LWB, WWB, and PWB.

Control variables: In accordance with the prior studies, Nurse's tenure, age, gender, education, marital status, fertility circumstance, and number of night shifts were included as control variables. These are generally associated with nurses' emotional exhaustion and well-being [38, 60].

### 3.3. Ethics

Ethical approval (MSB-202401) was granted by the institutional review board at the researchers' affiliated university. Participants were assured that their survey responses would be anonymous and used only for research purposes. The participants were free to withdraw at any time from participation in the study.

## 4. Results

Before proceeding to test the hypotheses, we performed a confirmatory factor analysis (CFA) to assess the study's measurement model. The measurement model was constructed by randomly creating three composite indicators for commuting stress, emotional exhaustion, and nurses' well-being, following the approach outlined by Little et al. [61]. The goodness of fit of the model was assessed using established evaluation methods proposed by Kline [62] and Hu and Bentler [63]. As indicated in Table 1, results indicate that the hypothesized three-factor model, which included commuting stress, emotional exhaustion, and nurses' well-being, demonstrated a better fit (*χ*^2^ = 135.26, *p* < 0.01, df = 41; CFI = 0.98; RMSEA = 0.078; SRMR = 0.04) as compared to alternative models. These results suggest that the study constructs are empirically distinct and that the study's measurement model is adequate.

Table 2 provides descriptive statistics and reliability estimates for the study's control and main variables along with the correlations among the variables. Coefficient alphas ranging from 0.93 to 0.96 demonstrated a high degree of internal reliability. Consistent with the study's hypotheses, commuting stress demonstrated a positive association with emotional exhaustion (*r* = 0.37, *p* < 0.01) and a negative association with nurses' well-being (*r* = −0.12, *p* < 0.05). Furthermore, emotional exhaustion was negatively associated with nurses' well-being (*r* = −0.45, *p* < 0.01). Correlations among potential control and main study variables were not substantial. Additionally, the average variances extracted (AVE) exceed the cutoff limit of 0.50, and the composite reliability (CR) values range from 0.931 to 0.964, surpassing the benchmark of 0.70 [64, 65]. These results provide evidence of the measurement model's discriminant and convergent validity.

### 4.1. Hypothesis Testing

The study hypotheses were tested using the PROCESS macro developed by Hayes [1]. The regression results, as shown in Table 3, reveal that commuting stress is positively related to emotional exhaustion (*B* = 0.30, *p* < 0.01) and negatively related to nurses' well-being (*B* = −0.07, *p* < 0.05). Additionally, emotional exhaustion is negatively related to nurses' well-being (*B* = −0.34, *p* < 0.05). Furthermore, the results indicate that commuting stress indirectly affects nurses' well-being through its impact on emotional exhaustion (*B* = −0.36, *p* < 0.01). These results support Hypotheses 1, 2, 3, and 4. Furthermore, the interaction between commuting stress and nurses' gender on emotional exhaustion was significant (*B* = 0.22, *p* < 0.05), thus supporting Hypothesis 5 and suggesting that the relationship between commuting stress and emotional exhaustion is greater for female nurses as compared to male nurses.

**Figure 2:**
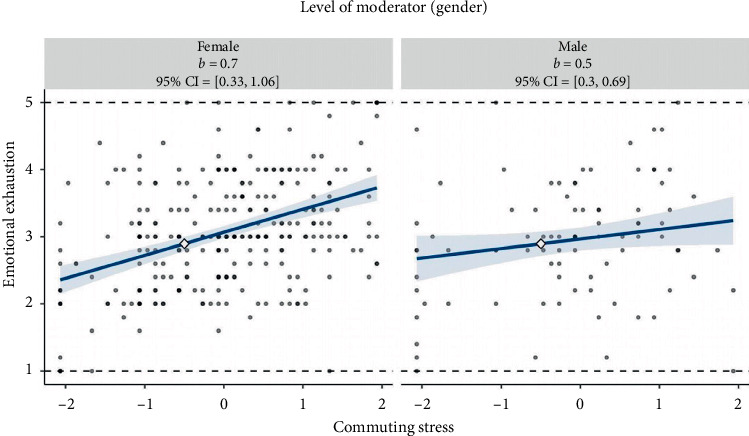
Simple slope–gender as a moderator. Two-way interaction of commuting stress and gender in the prediction of emotional exhaustion. Each graph illustrates the 95% confidence region (shaded area) calculated, the observed data (gray circles), and the upper and lower bounds of the outcome (dashed horizontal lines). The *x*-axis represents the full range of commuting stress. CI = confidence interval; PTCL = percentile.

**Figure 3:**
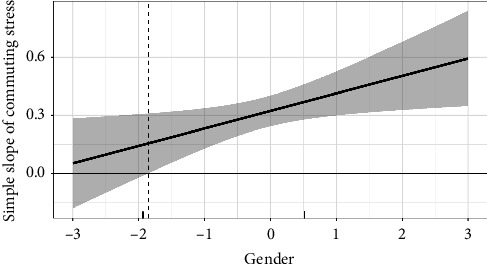
Marginal-effects plot with gender as moderator. The shaded regions indicate 95% confidence intervals. A marginal rug is provided on the horizontal axis to indicate observed data across the displayed range of gender. The vertical dashed lines represent the Johnson–Neyman values, which are the points on the gender where the confidence intervals stop crossing zero, indicating the slopes in these regions are statistically significant and nonzero.

To further confirm the research results, the study employed simple slope analysis and post hoc probing [66]. As shown in Figure 2, the slope is substantially steeper (i.e., significantly different at *p* < 0.05) for female nurses than for male nurses. Furthermore, as reflected in Figure 3, the marginal-effects plot demonstrates that the simple slope of commuting stress on emotional exhaustion is positive and significant when gender is −1.7 standard deviations or more away from the mean. Notably, 78.95% of observations in the sample are within this region. These additional analyses provide further support for the moderation effect proposed in Hypothesis 5. The steeper slope for female nurses, as well as the region of significance identified in the marginal-effects plot, suggests the relationship between commuting stress and emotional exhaustion is stronger for female nurses when compared to male nurses in this sample.

Regarding Hypothesis 6, the study's prediction was that the positive association between commuting stress and emotional exhaustion is moderated by gender, indicating that this relationship is stronger for female nurses compared to male nurses. As shown in Table 4, the results indicate that the negative relationship between commuting stress and nurses' well-being among female nurses is mediated by emotional exhaustion, and this indirect effect is statistically significant (*B* = −0.12, CI = −0.17, −0.08). Furthermore, the moderated mediation index was −0.08 (CI = −0.16, −0.01). Consequently, Hypothesis 6 received support from the data.

## 5. Discussion

This research tested a moderated mediation model to investigate the indirect effect of commuting stress on nurses' well-being, with emotional exhaustion as the mediating variable. The results yield empirical support for the hypothesized model. The results show a positive linkage between commuting stress and emotional exhaustion, as well as a negative association between commuting stress and nurses' well-being. Additionally, the study found that the relationship between commuting stress and nurses' well-being is mediated by emotional exhaustion, helping to explain this association. In addition, the findings suggest that nurse gender, particularly the female gender, amplifies the impact of commuting stress on emotional exhaustion. The moderated mediation analysis showed that nurse gender moderates the indirect effect of commuting stress on nurses' well-being through emotional exhaustion, with this indirect effect being stronger for female nurses as compared to male nurses. In summary, the findings indicate that the negative relationship between commuting stress and nurses' well-being is partially explained by increased emotional exhaustion, and this indirect relationship is greater for female nurses than for male nurses. These results underscore the importance of the gendered aspects of commuting and its implications for the well-being of nursing professionals.

### 5.1. Theoretical Implications

This study contributes significantly to the existing literature on nurses' commuting stress, emotional exhaustion, and well-being. First, previous research has shown that nursing work is highly demanding, imposing significant physical and psychological pressures on nurses. Consequently, numerous studies have examined how various occupational factors, including emotional exhaustion, influence nurses' job experiences and well-being [13]. However, empirical research investigating the spillover effects of nurses' experiences and encounters prior to entering the workplace has been limited [2, 6]. Specifically, commuting, which involves the additional stress aspect of traveling to the workplace, has a substantial impact on nurses' well-being. Drawing on the COR theory, our study identifies a key mechanism through which commuting stress affects nurses' emotions and well-being, providing empirical evidence that Chinese nurses experience the spillover effects of commuting stress. This study addresses the need to examine the generalizability of commuting-related influences among nurses in Eastern contexts, as previous research has primarily focused on Western contexts [6].

Second, previous research has demonstrated that the determinants of nurses' well-being encompass multiple levels. For instance, these determinants include physical condition, psychological states (such as depression and burnout), and individual-level factors like coping strategies or positive outlook, as well as group-level factors such as supervisor or coworker support, leadership style, and system capacity [15, 16, 67, 68]. The present study introduces new research perspectives to the examination of nurses' well-being, shedding light on the fact that, in addition to nurses' experiences within the workplace, their experiences outside the workplace can also impact their psychological state and well-being. Additionally, this study comprehensively measures nurses' well-being across three distinct domains, thereby offering relatively comprehensive and objective research findings compared to previous empirical studies on nurses' well-being.

Third, previous studies on nurses' emotional exhaustion have examined relevant antecedent variables, including job characteristics, emotional labor, and work schedule [69–71]. However, previous research has primarily focused on factors within the workplace context (e.g., the hospital setting) or job-related characteristics that impact nurses' emotional exhaustion, with limited attention given to potential influences from outside the workplace. The research findings reported here indicate that commuting stress, as a prework stressful event, depletes nurses' psychological and physiological resources, and subsequently, they must transition immediately to the highly demanding nature of nursing work. This is likely to contribute to nurses experiencing emotional exhaustion. Therefore, this study expands the research perspective on nurses' emotional exhaustion, demonstrating that the spillover effect from commuting stress serves as an additional antecedent of emotional exhaustion.

Finally, as mentioned previously, research has linked women's family roles to gendered commuting characteristics [22, 23, 54], and scholars argue that female nurses must balance their professional work responsibilities and household tasks. Previous empirical studies have not examined whether nurses' gender influences the spillover effect. In this study, the researchers argue and provide evidence suggesting that nurses' gender interacts with commuting stress, affecting their emotional exhaustion. This research is among the first to empirically examine the influence of gender differences on the commuting spillover effect among nurses. The results reveal the existence of gendered commuting characteristics among nurses, with female nurses being more susceptible to the detrimental effects of commuting stress on emotional exhaustion. The job characteristics of nursing contribute to a stronger spillover effect of commuting stress experienced by female nurses. The present findings not only expand the research on commuting among Chinese nurses but also validate the presence of gendered commuting characteristics within the Chinese nursing population.

### 5.2. Practical Implication

The present study's findings provide practical insights for developing interventions to support nurses in managing and mitigating the negative effects of commuting stress. Commuting stress is frequently overlooked within the nursing work environment, but the current study underscores its potential impact on nurses' emotional exhaustion and overall well-being. Since the stress of commuting to work is a potential factor leading to a nurse's diminished emotional resources and sense of well-being, thus, steps should be taken by healthcare organizations to put in place support systems for the mitigation of the negative effects of commuting. These steps could include, but not be limited to, a dedicated shuttle bus system for the nurses or financial subsidies to provide nurses with increased options and a greater sense of control over their commute. Other interventions might include scheduling changes, so the nurse is not required to commute during heavy commuter traffic times and/or provide stress relief measures upon arrival at the workplace. In the case where resources are available, worksite dormitory facilities or satellite worksites could be used to decrease the requirement for daily commutes. The purpose of these proposed interventions is to find a means of reducing the stress of commuting and improving the nurse's sense of well-being overall.

Since commuting can be stressful, this study highlights the crucial need to mitigate such stress and find ways to improve the overall sense of well-being for nurses. Organizations employing nurses would do well to use studies such as this one to push for workplace practices that improve a nurse's well-being. This might include the provision of counseling and self-care courses. Resources spent on the prevention of burnout will be recouped in multiples through the improved performance of the nurse. As the healthcare profession should know, “an ounce of prevention is worth a pound of cure.” As these research findings explain, gender difference is a contributing factor to the amount of stress encountered due to a commute. This is primarily due to social contexts positioning female nurses to face more difficult commutes. Knowing this should shape the approaches to interventions such that the stress female nurses face will be addressed. These programs may include stress management workshops, workplace spirituality sessions, physical activity initiatives, or providing access to relaxation and meditation spaces within the workplace. Workplace interventions might range from the provision of female focused but not exclusive workshops on spirituality, importance of exercise to the creation of dedicated spaces on site for meditation and relaxation.

Recognizing the degree to which this research highlights the stress of a commute, it also highlights the need for organizations to include the issue of stress due to commute into their occupational health and safety policies. When commuting stress is identified on a level equal to other occupational hazards, the ground is set for changes to be made such that nurses experience a healthier workplace. Suggested guidelines should consider issues related to commuting to work as described in this paper. At the same time, it is important that managers be trained to recognize the issues and stress so they can consistently apply and, when necessary, revise the policy. Well-trained managers will make sure the policies are relevant as well as meeting the needs of the nurses. A scheduled evaluation process can assess the effectiveness of issues including parking regulations, transportation subsidies, scheduling of work hours, and other safety protocols. Recognizing the unique challenges faced by female nursing staff, the objective will be to tailor policies to ease the stress faced by commuting to work.

### 5.3. Limitations and Future Research

The findings of the current study are subject to certain limitations, such as relying on self-reporting by the nurses, and these same limitations introduce opportunities for further research. Collecting data at three time points (see [56]) was performed in an attempt to reduce the limitation of self-reporting. Future research on this topic will be better served through a longitudinal design in order to more definitively assess causal relationships. The exclusive context being in Chinese hospitals presents a second limitation for the study. Additional research will need to be carried out in different cities and countries where public and private transportation systems are in play [2, 22, 23]. It would also be helpful to explore different social expectations for female nurses based on different cultures and countries. Replication studies can strengthen the generalizability of the findings reported here and provide a deeper understanding of the impact of commuting stress on nurses' emotional exhaustion and well-being. Third, this study focused only on the joint effect of commuting stress and gender on nurses. Other personal characteristics (e.g., personality traits) and job characteristics [2] may also influence the spillover effect of commuting stress for nurses. Future research should explore the joint effects of these factors on nurses' psychological states and behaviors.

## 6. Conclusion

In closing, by highlighting the spillover effects of commuting stress on nurses' well-being through emotional exhaustion in a Chinese context, the research described here makes important and compelling contributions relative to the nursing literature. Our findings suggest not only that commuting stress can have deleterious effects on nurses' emotional exhaustion and well-being, which in turn may negatively affect the quality of care and patient well-being, but also that these harmful outcomes may be especially pronounced for female nurses. These results are particularly exciting because they may help to positively and proactively shape nursing practice and policies in the healthcare industry, while also inspiring future research aimed at further enhancing nurses' well-being as well as the well-being of other healthcare professionals.

## Figures and Tables

**Table 1:** Confirmatory factor analysis of measures.

Model	Factors	*χ* ^2^	Δ*x*^2^	*df*	RMSEA	CFI	SRMR
Baseline model	Three	135.26		41	0.078	0.98	0.04
Alternative Model 1	Two	1621.86	1486.6⁣^∗∗^	43	0.31	0.64	0.26
Alternative Model 2	Two	1719.24	1583.98⁣^∗∗^	43	0.32	0.61	0.24
Alternative Model 3	One	3117.67	2982.41⁣^∗∗^	44	0.43	0.29	0.32

*Note:* Model 1: Commuting stress and emotional exhaustion were combined into one factor. Model 2: Commuting stress and nurses' well-being were combined into one factor. Model 3: All three variables were combined into one factor.

⁣^∗∗^*p* < 0.01.

**Table 2:** Correlations and descriptive statistics.

	M	SD	1	2	3	4	5	6	7	8	9	10	Cronbach's α	Composite reliability	Average variance extracted
1. Tenure	13.73	9.65	—												
2. Age	36.22	9.62	0.91⁣^∗∗^	—											
3. Education	2.98	0.63	0.11⁣^∗^	0.19⁣^∗∗^	—										
4. Marital status	1.76	0.50	0.60⁣^∗∗^	0.59⁣^∗∗^	0.10	—									
5. Fertility circumstance^a^	1.70	0.46	0.62⁣^∗∗^	0.62⁣^∗∗^	0.13⁣^∗^	0.87⁣^∗∗^	—								
6. Number of night shifts	2.85	1.59	−0.36⁣^∗∗^	−0.34⁣^∗∗^	−0.07	−0.24⁣^∗∗^	−0.27⁣^∗∗^	—							
7. Gender^b^	0.79	0.41	−0.12⁣^∗^	−0.22⁣^∗∗^	−0.28⁣^∗∗^	−0.07	−0.10	0.02	—						
8. Commuting stress	3.07	1.03	0.04	0.01	0.04	−0.00	0.03	0.08	0.07	—			0.957	0.956	0.69
9. Emotional exhaustion	3.05	0.85	−0.05	−0.08	0.05	−0.14⁣^∗∗^	−0.12⁣^∗^	0.15⁣^∗∗^	0.07	0.37⁣^∗∗^	—		0.926	0.931	0.73
10. Nurses' well-being	3.36	0.66	0.08	0.08	0.05	0.10⁣^∗^	0.11⁣^∗^	−0.18⁣^∗∗^	−0.03	−0.12⁣^∗^	−0.45⁣^∗∗^	—	0.963	0.964	0.60

*Note: N* = 380; reliability coefficients are reported on the diagonal.

⁣^∗^*p* < 0.05.

⁣^∗∗^*p* < 0.01.

^a^0 = no child, 1 = one child, 2 = two children, 3 = more than two children.

^b^0 = male, 1 = female.

**Table 3:** Regression results of PROCESS.

Path estimated	Emotional exhaustion		Nurse well-being		
Constant	2.40⁣^∗∗^	3.12⁣^∗∗^	4.38⁣^∗∗^	3.51⁣^∗∗^	4.44⁣^∗∗^
Commuting stress	0.30⁣^∗∗^	0.30⁣^∗∗^	0.03	−0.07⁣^∗^	
Emotional exhaustion			−0.36⁣^∗∗^		−0.34⁣^∗∗^
Gender		0.14			
Commuting stress ∗ Gender		0.22⁣^∗^			
*R* ^2^	0.17⁣^∗∗^	0.19⁣^∗∗^	0.23⁣^∗∗^	0.05⁣^∗∗^	0.20⁣^∗∗^

*Note: N* = 380. The values in the table are path estimates from the estimated model. Unstandardized regression coefficients are reported. Bootstrap sample size = 5000.

⁣^∗^*p* < 0.05.

⁣^∗∗^*p* < 0.01.

**Table 4:** Regression results for conditional indirect effect.

Predictor	*B*	SE	*t*	*p*
*Emotional exhaustion*				
Constant	3.12	0.34	9.14	0.00
Commuting stress	0.30	0.04	7.60	0.00
Gender	0.14	0.11	1.29	0.20
Commuting stress × gender	0.22	0.09	2.43	0.02

*Nurses' well-being*				
Constant	4.48	0.28	16.16	0.00
Commuting stress	0.03	0.03	1.08	0.28
Emotional exhaustion	−0.36	0.04	−9.22	0.00

**Gender**	**Boot indirect effect**	**Boot SE**	**BootLL 95% CI**	**BootUL 95% CI**

*Conditional indirect effect*				
Female	−0.12	0.03	−0.17	−0.08
Male	−0.05	0.04	−0.10	0.02

*Index of moderated mediation (difference between conditional indirect effects)*				
Gender	−0.08	0.05	−0.16	−0.01

*Note: N* = 380. Unstandardized regression coefficients are reported. Bootstrap sample size = 5000.

Abbreviations: CI = confident interval, LL = lower limit, UL = upper limit.

## Data Availability

The data that support the findings of this study are available on request from the corresponding author. The data are not publicly available due to privacy or ethical restrictions.
